# Derivation of a frailty index from the interRAI acute care instrument

**DOI:** 10.1186/s12877-015-0026-z

**Published:** 2015-03-18

**Authors:** Ruth E Hubbard, Nancye M Peel, Mayukh Samanta, Leonard C Gray, Brant E Fries, Arnold Mitnitski, Kenneth Rockwood

**Affiliations:** Centre for Research in Geriatric Medicine, The University of Queensland, Brisbane, QLD Australia; QIMR Berghofer Medical Research Institute, Brisbane, QLD Australia; Geriatrics Center, Department of Internal Medicine and School of Public Health, University of Michigan, Ann Arbor, MI USA; Department of Medicine, Dalhousie University, Halifax, NS Canada

**Keywords:** Frailty index, Inpatients, Aged, 80 and over, Geriatric assessment

## Abstract

**Background:**

A better understanding of the health status of older inpatients could underpin the delivery of more individualised, appropriate health care.

**Methods:**

1418 patients aged ≥ 70 years admitted to 11 hospitals in Australia were evaluated at admission using the interRAI assessment system for Acute Care. This instrument surveys a large number of domains, including cognition, communication, mood and behaviour, activities of daily living, continence, nutrition, skin condition, falls, and medical diagnosis.

**Results:**

Variables across multiple domains were selected as health deficits. Dichotomous data were coded as symptom absent (0 deficit) or present (1 deficit). Ordinal scales were recoded as 0, 0.5 or 1 deficit based on face validity and the distribution of data.

Individual deficit scores were summed and divided by the total number considered (56) to yield a Frailty index (FI-AC) with theoretical range 0–1. The index was normally distributed, with a mean score of 0.32 (±0.14), interquartile range 0.22 to 0.41. The 99% limit to deficit accumulation was 0.69, below the theoretical maximum of 1.0.

In logistic regression analysis including age, gender and FI-AC as covariates, each 0.1 increase in the FI-AC increased the likelihood of inpatient mortality twofold (OR: 2.05 [95% CI 1.70 – 2.48]).

**Conclusions:**

Quantification of frailty status at hospital admission can be incorporated into an existing assessment system, which serves other clinical and administrative purposes. This could optimise clinical utility and minimise costs.

The variables used to derive the FI-AC are common to all interRAI instruments, and could be used to precisely measure frailty across the spectrum of health care.

## Background

The care of older people with multiple co-morbidities is a core remit of our acute hospitals, yet the health care system is better designed to meet the needs of younger, fitter patients with single system problems. The management of a 50 year old man with an acute myocardial infarction, for example, is underpinned by a wealth of research data: his investigations, pharmacotherapy and optimal nursing environment can each be guided by algorithm. Although Comprehensive Geriatric Assessment results in better outcomes [1], decisions for complex older inpatients are often undertaken without the benefit of a strong evidence base. This weak evidence base results in frustration and feelings of inadequacy for the treating doctor [2]. More importantly, it can result in inappropriate care. Some older people are subject to futile and distressing treatment at the end of their lives [3]; others are denied potentially beneficial interventions on the basis of their chronological age alone [4].

A measurement of frailty status for older inpatients may help target their care more appropriately. Frailty identifies individuals with a diminished capacity to compensate effectively for external stressors [5] yet it has, to date, proven challenging to quantify in clinical practice. There are two main approaches to defining and measuring frailty [6]. The deficit model consists of summing an individual’s number of impairments and conditions to create a Frailty index [7], while the frailty phenotype is a clinical syndrome in which three or more of the following criteria are present: unintentional weight loss, self-reported exhaustion, weakness (grip strength), slow walking speed, and low physical activity [8]. The prevalence of frailty varies widely according to the assessment instrument used [9]; many inpatients are unable to complete the performance based tests integral to phenotypic measures [10]; counting accumulated deficits has been criticised as too complex for initial evaluation [11]. Although some reports have evaluated a Frailty index based on deficit accumulation in patients admitted to acute care [9,12,13] each is a study of fewer than 800 people from a single hospital site.

Here, we describe the derivation of a Frailty index from the interRAI assessment system for Acute Care (the FI-AC). We consider how deficits were chosen for inclusion and how cut-points were established for ordinal variables. The properties of the FI-AC, particularly its distribution, limit and relationship to chronological age and mortality, are reported and compared to the usual characteristics of a Frailty index.

## Methods

### Study sample

Our sample comprised 1418 patients aged 70 and older admitted to eleven acute care hospitals in Queensland and Victoria, Australia. The sites ranged from small secondary care centres (with 120 – 160 beds, n = 2) and rural hospitals (250 – 280 beds, n = 2) to metropolitan teaching facilities (300 – 450 beds, n = 4) and major tertiary referral centres (>650 beds; n = 3). Recruitment took place between July 2005 and May 2010 as part of three separate cohort studies, described in detail elsewhere [14-16]. Patients were excluded if they were admitted to coronary or intensive care units, received palliative care only, or were transferred out of general medical units within 24 hours of admission to inpatient wards.

### Measurement tool and outcome measures

The interRAI assessment system for Acute Care (interRAI AC) tool has been specifically developed for use in the acute setting, to support the comprehensive geriatric assessment of older inpatients [17,18]. Trained nurse assessors gather information about the patient’s physical, cognitive and psycho-social functioning, based on observations of patients during their first 24 hours in the ward. All available sources of information, including the patient, medical/nursing/allied health staff, the medical record and family members, are utilized to complete the interRAI AC instrument. The information is systematically interpreted, providing clinical profiles and generating problem lists and suggestions for care planning and facilitating the centralization of data [19]. The clinical items have been shown to provide a valid measure of function in frail patients [20] and have very good inter-rater reliability, with kappa scores for median observed agreement of 0.89, and greater than 0.80 for 83% of items [21].

### Selecting candidate deficits

All deficits were taken from data collected as part of the interRAI AC assessment. Previous work among community-dwellers suggests that variables can be considered as *deficits* and contribute to a Frailty index if they satisfy five criteria [22]:

1. **Variables must be associated with health status.** To assist in evaluation and care planning for older inpatients, the interRAI AC collects information on each patient’s social and physical living environment as well as legal guardian status and advanced directives. This data were not included in the Frailty index. Similarly, interventions such as chemotherapy, renal dialysis, ventilatory support and nasogastric or parenteral feeding are carefully recorded in the interRAI assessment. Although some of these treatments and procedures do increase risk of adverse outcomes, they are not reflective of patients’ health status and were not considered as deficits.
2. **The deficits that make up a Frailty index must cover a range of systems.** The interRAI AC instrument screens a large number of fields: cognition, communication, mood and behaviour, functional status, continence, disease diagnoses, health conditions (falls, pain, shortness of breath and fatigue), nutritional status, skin condition and medications. All domains were represented in the FI-AC.
3. **A deficit’s prevalence should generally increase with age.** Some adverse conditions, such as cancer, decrease in prevalence at very advanced ages due to survivor effects [23] but are still clearly age-related deficits and were included in this FI. Note too that although psychological distress tends to peak in middle age [24], depression and anxiety make a unique contribution to adverse outcomes in older people and were considered important contributory deficits.
4. **The chosen deficits must not saturate.** Problems that have a very high prevalence in old age should not be included in a Frailty index as they would not contribute to the stratification of health status. Presbyopia (age-related lens changes resulting in problems with accommodation) is an example for community-dwellers as this condition begins at aged 40 years and eventually affects everyone [25]. In the interRAI dataset, no deficits were ubiquitous. Redundant questions were excluded during development of the interRAI instrument, when the psychometric properties of each item were examined [17].
5. **If a single frailty index is to be used serially on the same people, the items that make up the Frailty index need to be the same across iterations.** The interRAI suite includes tools to assess patients with chronic illness, disability and mental health problems across different settings (home, hospital, hospice, long term care facilities) [18]. The current ten instruments comprise the same core data items plus optional items specific to particular situations. We chose to derive the FI-AC from only core items, affording the opportunity to track the health status of patients across care settings by calculating the Frailty index from different instruments.

### Coding of individual variables

All binary variables were recoded, using the established convention that ‘0’ indicated the absence of the deficit and ‘1’ the presence of a deficit. For ordinal and continuous variables, coding was based on face validity using clinical judgement and according to distribution of the data [22]. Consensus on coding was reached in discussion and correspondence among authors. For example, the Cognition section of the interRAI-AC interrogates acute change in mental status from the person’s usual functioning. This is defined as restlessness, lethargy, being difficult to arouse or displaying altered environmental perception [26]. The assessor is advised that accurate assessment requires conversations with staff or others who have direct knowledge of the person’s behaviour over the past 24 hours. The interRAI coding of no = 0; yes = 1 was directly translated to the absence or presence of a deficit. For some cognitive domains, such as being easily distracted, the interRAI-AC distinguishes between “Behaviour not present” = 0, “Behaviour present, consistent with usual functioning” = 1 and “Behaviour present, different from usual functioning” = 2. For these variables, the presence of the behaviour, regardless of its duration, was coded as 1 deficit.

For variables that included three potential responses (e.g. ‘No pain” = 0, “Present but not exhibited in the last 24 hours” = 1, “Exhibited in the last 24 hours” = 2), the intermediate value was coded as 0.5. Evidence from population-based longitudinal studies of community dwellers (N = 41,527) suggests that grading the severity of a deficit rather than dichotomising does not improve the performance of the index in predicting mortality [27]. However, as this has yet to be confirmed in an inpatient population, we chose to exploit the granularity of available data.

Within the interRAI-AC, measures of functional status are based on the patient’s self-care performance over the preceding 24 hours. These are recorded by the nurse assessor on a scale from 0 through 6, with a response of 8 if the activity did not occur. These ordinal scales do not manifest clear gradations in relation to deficit cut-points, hence recoding of variables was based on clinical judgement. The extremes of the scales were straightforward: the interRAI value of 0 = “independent” translated directly to 0 = absence of the deficit, while 6 = “total dependence” was coded as 1 = full expression of the deficit. An interRAI score of 1 = “independent, set up help only” is used when an article or device is provided or placed within reach. Examples include providing a wash basin and grooming articles for personal hygiene or handing the person a cane for walking. Since no physical assistance or supervision is provided, and placing an article in reach may be an artefact of the hospital environment, scores of 1 were also coded as no deficit. For each functional domain, interRAI convention is to score 2 if “supervision” is required for an activity. The requirement for a nurse to remain nearby to watch over a patient while they use the toilet, for example, reflects a step-wise increase in care needs compared to set-up help only. “Supervision” was therefore coded as 0.5.

Scores on the interRAI of 3 through 5 represent increasing levels of dependency:

- 3 = limited assistance – guided manoeuvring of limbs, physical guidance without taking weight
- 4 = extensive assistance – weight-bearing support (including lifting limbs) by one helper where person still performs 50% or more of subtasks
- 5 = maximal assistance – weight-bearing support (including lifting limbs) by two or more helpers or weight-bearing support for more than 50% of subtasks

While it is axiomatic that patients needing maximal assistance are more reliant than those needing limited assistance, the gradation from 2 to 3 represents the transition to dependence upon another person to complete a functional task. Since it is, arguably, this transition that impacts discharge planning more than the intensity of required support, we assigned 1 deficit for scores of 3, 4 or 5.

To complete coding of functional domains, in view of the association between failure to complete performance-based measures and adverse outcomes [26], an interRAI score of 8 was also coded as 1 full deficit.

The coding of deficits within the domains of communication and vision, mood and behaviour, continence, pain, oral and nutritional status and skin was based on both clinical judgement and on the distribution of the data. For example, in the assessment of hearing, we considered coding only “severe difficulty” or “no hearing” as a full deficit. However, the prevalence of a deficit so defined would have been low, at only 2.9% of the sample. “Moderate difficulty” (corresponding to “problem hearing normal conversation; requires quiet setting to hear well”) applied to 8.3% of our patients and retains strong face validity as an age-associated problem with an adverse effect on health status. Hence, the coding for hearing was 0 = adequate: no deficit, 1 = minimal difficulty: 0.5 deficit; ≥ 2: 1 deficit. Congruent with the principle applied to functional domains, failure to complete cognitive assessments is associated with adverse outcomes [28] and patients who could not or would not respond regarding their interest in things, anxiety or sadness were allocated a full deficit for each question.

Each disease diagnosis (whether 1 = primary diagnosis; 2 = disease present, receiving active treatment or 3 = disease present, monitored but no active treatment) was counted as one deficit. At the time of data collection, the interRAI-AC was structured to allow the documentation of up to 10 disease diagnoses and this maximal number was recorded for 126 patients (8.9% of the cohort), with a normal distribution around a mean and median value of 6. Analysis of interRAI Home Care data (N = 351) in which up to 31 diagnoses can be recorded revealed a normal distribution with a mean of 6 and a range from 0 (0.6%) up to 16 (0.3%). For the patients with 10 or more disease diagnoses, the mean was 11.7. Since the interRAI-AC subgroup recorded as having 10 diagnoses was likely to include patients with between 10 and 15 diagnoses, the denominator accounted for up to 15 disease diagnoses and those at the ceiling of 10 were recoded as 12. The interRAI-AC has subsequently been amended to enable capture of up to 15 diagnoses. Whether more precise quantification for those with multiple morbidities has a clinically meaningful impact on FI-AC scores is the focus of ongoing enquiries.

In older people, the association between body mass index (BMI) and adverse outcomes shows a U-shaped curve [29] and increasing numbers of medications predict both disability and mortality [30]. BMI, calculated from height (cm) and weight (kg), was therefore coded as 0 deficit for those with a BMI of 20 – 30, 1 deficit if BMI < 20 or >30. Having observed that patients were prescribed between 0 and 25 regular medications per day, we allocated up to 4 deficits for increasing polypharmacy (≤4 medications = 0 deficit; 5 – 9 medications = 1 deficit; 10 – 14 medications = 2 deficits; 15 – 19 medications = 3 deficits; ≥ 20 medications = 4 deficits).

### Analysis

We investigated the distribution of FI-AC, its relationship with age and variance in the slope of FI-AC by conducting various statistical analyses. A histogram was used to confirm the approximate normality of the FI-AC. A fitted linear regression model was used to identify the relationship between average FI-AC and age. Due to the small numbers of patients aged ≥ 100 years (N = 2), centenarians were excluded from this model. The rate of accumulation of deficits was calculated by evaluating the slope of the line. To observe the upper limit of the FI-AC, the 99th percentiles were also plotted against age and a linear line fitted to these data. A flattening of this line (i.e. a parallel line to age-axis) would suggest the rate of deficit accumulation reaches a limit with increasing frailty.

A sampling procedure was adopted to extract the information about variability of the slope of FI-AC and to evaluate the impact of a given variable on the FI-AC. We randomly selected 80% of the variables without replacement and calculated FI-AC for each sample. The average FI-AC was plotted against age and the slope and intercept recorded. This procedure was repeated 1000 times to generate 1000 samples which were then evaluated.

### Ethics

Personal or proxy consent was obtained in writing by each participant prior to commencement of the studies. Ethical approval was granted from the human research and ethics committee of each participating hospital and University of Queensland medical research ethics committee.

## Results

The study population included 1418 patients with a mean (SD) age of 81.0 (6.8) years; the majority were female (n = 780; 55%). The median (interquartile range) length of stay in acute care was 6 (4–11) days and the majority (87.2%) were admitted to hospital from the community. Characteristics of the population are shown in Table 1.

**Table 1:** **Characteristics of the study population**

**Characteristic**	**N = 1418**
Age (years) mean (SD)	81.0 (6.8)
Females n (%)	780 (55.0%)
Length of stay (days) median (IQR)	6 (4–11)
Admitted from:	
- Community	1236 (87.2%)
- Other hospitals	11 (0.8%)
- Residential aged care (low care)	85 (6.0%)
- Residential aged care (high care)	85 (6.0%)
Discharged to:	
- Community	919 (64.8%)
- Post-acute care (including rehabilitation)	234 (16.5%)
- Residential aged care (low care)	83 (5.9%)
- Residential aged care (high care)	124 (8.8%)
- Died in hospital	57 (4.0%)
Principal diagnosis on admission^a^:	
- Circulatory system diseases	277 (19.6%)
- Respiratory diseases	235 (16.6%)
- Injuries	142 (10.1%)
- Symptoms and signs	129 (9.1%)
- Diseases of the digestive system	114 (8.1%)
- Disorders of the genitourinary system	97 (6.9%)
- Neoplasms	87 (6.2%)

Notes.

SD: Standard Deviation.

IQR: Interquartile Range.

^a^Principal diagnosis based on International Statistical Classification of Diseases and Related Health Problems, Tenth Revision (ICD-10).

### Construction and characteristics of the frailty index-acute care

39 variables that met all Frailty index criteria were chosen. 37 of these translated directly into 37 potential deficits with 15 potential deficits allocated for “disease diagnoses” and 4 for “number of regular medications per day”. Hence the total number of potential deficits was 56.

An FI-AC could be derived for 100% of the cohort.1269 patients (89.5%) had complete data across all domains and their FI-AC was derived by summing the deficits present and dividing by 56. 149 patients had at least 1 missing item, totalling 298 missing data points (0.5% of the 55302 total – 39 variables for 1418 patients). For these patients, the denominator was reduced accordingly (e.g. to 55 for 111 patients (7.8%) in whom 1 item was missing).

**Figure 1:**
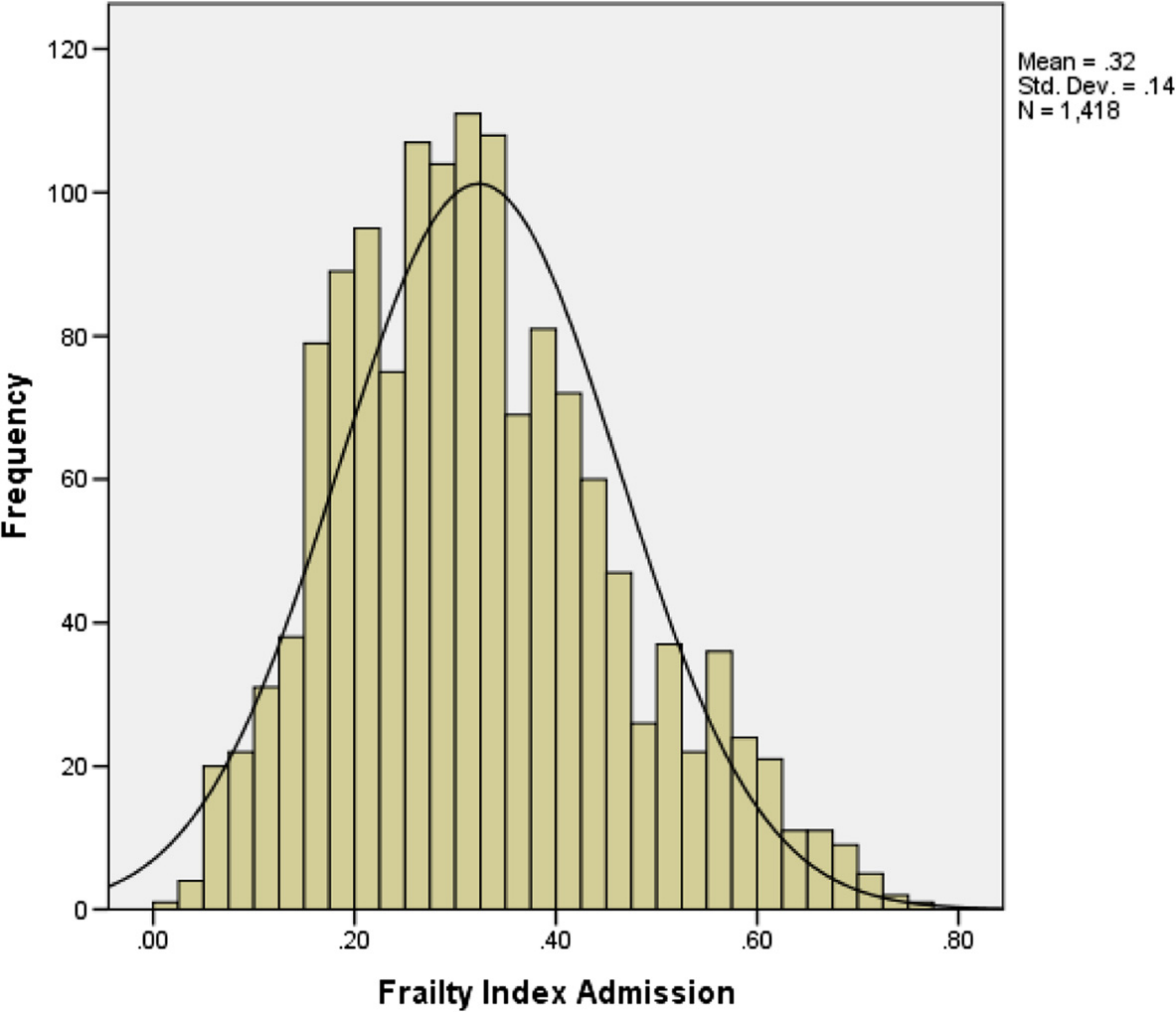
**Distribution of the frailty index-acute care.**

The index was normally distributed, with a mean score of 0.32 (±0.14), interquartile range 0.22 to 0.41 (Figure 1). The 99% limit to deficit accumulation was below the theoretical maximum of 1.0 at 0.69.

**Figure 2:**
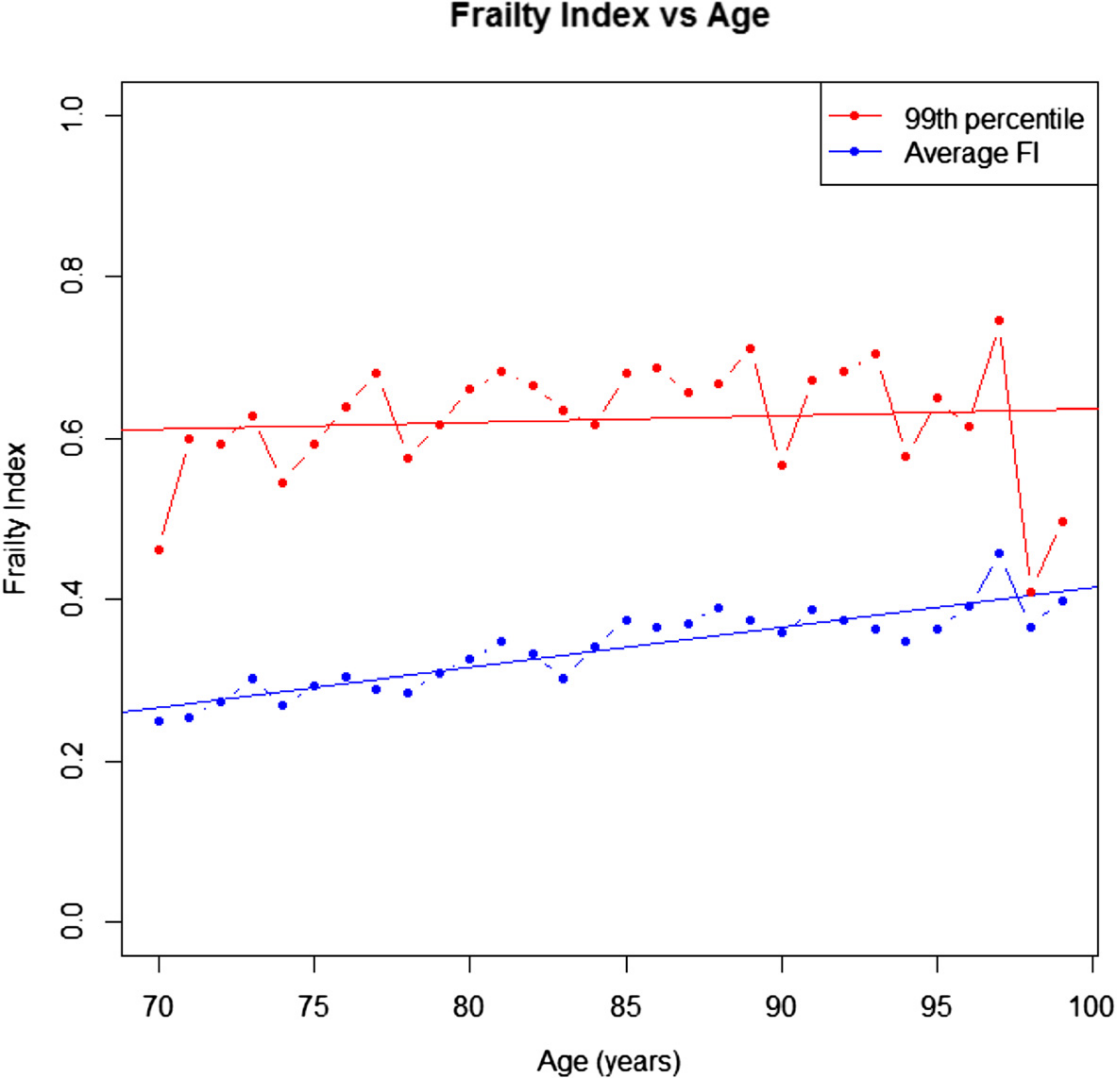
**Frailty index-acute care versus age plot.** Legend: Average FI-AC (blue) and the 99th percentile (red) are shown against age. Best fit regression lines are overlaid to illustrate no accumulation of deficits in the 99th percentile (red) and 0.5% deficit accumulation per year in the average FI-AC (blue).

In relation to age, the average slope of the deficit accumulation line was 0.005 [95% CI (0.004, 0.006)] (Figure 2). For those at the upper limit of the Frailty index (99th percentile) the average slope of deficit accumulation was 0.0008 [95% CI (−0.002, 0.004)], suggesting that for the frailest older people, there was little evidence of any relationship between age and deficit accumulation (Figure 2).

**Figure 3:**
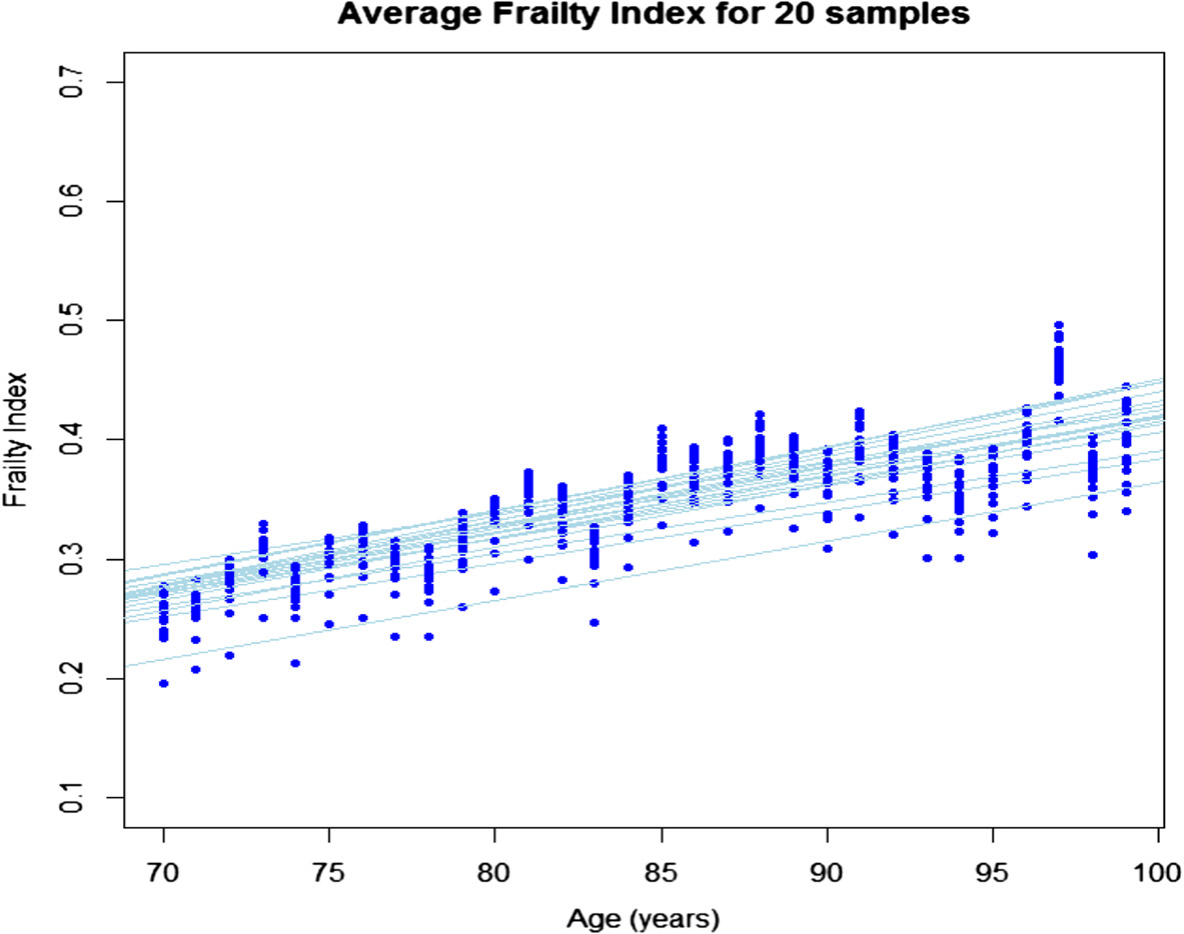
**Average Frailty index for 20 samples.** Legend: The Frailty index was created 1000 times, each time randomly picking 80% of the variables of the index. Twenty randomly (out of 1000) selected experimental and best fit regression lines of the average Frailty index are shown here.

Random sampling of the FI-AC (Figure 3) showed that when 80% variables were sampled, the difference in slopes was negligible, which indicated there was little sensitivity as to which variables were included in FI-AC construction. The differences in the intercepts of the fitted lines also illustrated the variance in the slope of the FI-AC.

### Mortality in relation to the frailty index- acute care

The FI-AC was associated with in-hospital mortality. In a logistic regression analysis including age, gender and FI-AC as covariates, each 0.1 increase in FI-AC increased the likelihood of inpatient mortality twofold (OR: 2.05 [95% CI 1.70 – 2.48]).

## Discussion

For older people admitted to hospital, a Frailty index can be derived from data collected using the interRAI Acute Care instrument. It can be calculated for all inpatients, even those who are bed-bound and highly dependent. The FI-AC conforms to the usual characteristics of a Frailty index. While a gamma distribution is well-described in community-dwellers [31], a normal distribution is expected here in view of the more homogenous, more unwell population [32]. The slope in relation to age was indistinguishable from zero in those at the upper limit of frailty [33] and the age independent limit to frailty was submaximal, at 0.69 [34]. The association of the FI-AC with in-hospital mortality, independent of chronological age, supports its predictive validity as a measure of health status.

Many tools and instruments are used in the assessment of older people admitted to hospital, often with a high level of information duplication. Instruments vary widely between settings and use different terminologies and scoring systems; many have not been validated in older people. These “first-generation” screening tools usually comprise assessment of a single domain (such as nutritional status) triggered by clinical impression (perhaps concern about weight loss) by disciplines with domain-specific expertise (the dietician) [35]. In contrast, the interRAI AC instrument examines a large number of domains, which affords the opportunity to derive a Frailty index at hospital admission. While the interRAI AC does not yet have widespread international endorsement, it does have more uptake than any other omnibus assessment system, already being integral to inpatient care across many secondary and tertiary settings. The derivation of an FI-AC from this instrument does not depend on the collection of any additional data and hence this measure of frailty could potentially be implemented into routine clinical practice without the need for major financial investment, the employment of research personnel or additional examinations for the patient. A Frailty index based on a standardized interRAI AC may therefore provide a feasible and highly cost-effective means of stratifying the health status of older adults who present to hospital.

Our results can be contextualised with other inpatient populations. A Frailty index derived from a one-page Comprehensive Geriatric Assessment form (FI-CGA) has been previously investigated by our group: for example, in 178 patients (mean 81 years) admitted with hip fracture in Cardiff, South Wales [13] and in 752 older people (mean 84 years) admitted to medical units in Buffalo, USA [12]. Mean FI-CGAs [of 0.34 and 0.38 respectively] were closely comparable to the mean FI-AC, particularly taking into account the different ages of these cohorts.

Our data must be interpreted with caution. Patients were recruited from Australian hospitals only, yet our sample was moderately large, and the inclusion of hospitals with different sizes and designations increases the generalizability of our findings. Here, data were collected as part of research studies. The completeness and rigour of interRAI-AC data in routine clinical practice, and the validity of the resulting FI-AC, are the focus of current enquiries. Note that when a Frailty index from the interRAI Home Care (HC) instrument was investigated in a large operational dataset [36], each patient’s FI could be calculated.

The interRAI-AC is compatible with other interRAI assessment systems used extensively across Europe and North America, New Zealand and Singapore. In contrast to previous work [36], this FI comprises only core data items, providing the capacity to calculate the Frailty index across different systems and settings of health care. Tracking the health status of patients across care settings would have benefits at the individual level; describing the health of populations with a parsimonious and uniformly understood measure would have benefits at an epidemiological level. For example, higher mortality in residential aged care facilities in one country compared to another could be explained if the mean core-FI of admitted patients was significantly different but could otherwise trigger careful systems review.

## Conclusion

Quantification of frailty status at hospital admission can be incorporated into an existing assessment system, which serves other clinical and administrative purposes. This could optimise clinical utility and minimise costs.

The variables used to derive the FI-AC are common to all interRAI instruments, and could be used to precisely measure frailty across the spectrum of health care.
